# A Human Brain Microphysiological System Derived from Induced Pluripotent Stem Cells to Study Neurological Diseases and Toxicity

**DOI:** 10.14573/altex.1609122

**Published:** 2016-11-24

**Authors:** David Pamies, Paula Barrera, Katharina Block, Georgia Makri, Anupama Kumar, Daphne Wiersma, Lena Smirnova, Ce Zhang, Joseph Bressler, Kimberly M. Christian, Georgina Harris, Guo-li Ming, Cindy J. Berlinicke, Kelly Kyro, Hongjun Song, Carlos A. Pardo, Thomas Hartung, Helena T. Hogberg

**Affiliations:** 1 Center for Alternatives to Animal Testing; 2 Department of Neurology; 3 Division of Neuroimmunology; 4 Institute for Cell Engineering; 5 Hugo Moser Institute at the Kennedy Krieger; 6 The Solomon Snyder Department of Neuroscience; 7 Wilmer Eye Institute, Johns Hopkins University, Baltimore, MD, USA; 8 US Army Edgewood Chemical Biological Center, Aberdeen Proving Ground, MD, USA; 9 University of Konstanz, Konstanz, Germany

**Keywords:** 3D culture, CNS, myelination, microphysiological system, brain

## Abstract

Human *in vitro* models of brain neurophysiology are needed to investigate molecular and cellular mechanisms associated with neurological disorders and neurotoxicity. We have developed a reproducible iPSC-derived human 3D brain microphysiological system (BMPS), comprised of differentiated mature neurons and glial cells (astrocytes and oligodendrocytes) that reproduce neuronal-glial interactions and connectivity. BMPS mature over eight weeks and show the critical elements of neuronal function: synaptogenesis and neuron-to-neuron (e.g., spontaneous electric field potentials) and neuronal-glial interactions (e.g., myelination), which mimic the microenvironment of the central nervous system, rarely seen *in vitro* before. The BMPS shows 40% overall myelination after 8 weeks of differentiation. Myelin was observed by immunohistochemistry and confirmed by confocal microscopy 3D reconstruction and electron microscopy. These findings are of particular relevance since myelin is crucial for proper neuronal function and development. The ability to assess oligodendroglial function and mechanisms associated with myelination in this BMPS model provide an excellent tool for future studies of neurological disorders such as multiple sclerosis and other demyelinating diseases. The BMPS provides a suitable and reliable model to investigate neuron-neuroglia function as well as pathogenic mechanisms in neurotoxicology.

## 1 Introduction

There is a lack of mechanistic understanding of processes related to (developmental) neurotoxicity (Smirnova et al., 2014; Schmidt et al., 2016) and neurological disorders, partly due to limited representative models of humans. Animal-based models have poor predictivity for human health (Hartung, 2013, 2008), and do not always mimic human pathology. More than 90% of all drugs fail clinical trials despite extensive animal testing (Hartung, 2013), in part because animal studies do not reflect human physiology and inter-individual differences. Simple *in vitro* systems do not represent complex physiology and organ function (Hartung, 2007), especially that of the brain. This illustrates a critical need for better models for drug development, the study of disease, bioengineering and toxicological testing.

Some attempts to generate more complex organotypic cultures or microphysiological systems (MPS) have resulted in physiological multicellular 3D co-culture models with the ability to simulate functional parts of the brain (Lancaster et al., 2013; Kadoshima et al., 2013). Furthermore, the discovery of induced pluripotent stem cells (iPSC) and protocols to differentiate them into various cell types has boosted the development of new human *in vitro* models (Takahashi et al., 2007). iPSC from healthy or patient donors (Nieweg et al., 2015; Raitano et al., 2015) used in MPS promise more human-representative models, such as brain organoids that have been able to recapitulate features of human cortical development (Lancaster et al., 2013; Kadoshima et al., 2013). These complex systems represent novel tools for the study of biological mechanisms in the CNS. However, their application to drug screening and chemical testing has limitations. Some require elaborate and complex protocols and the marked variations in number of cells and composition of organoids may negatively impact reproducibility of cell differentiation. Moreover, necrosis can occur at the center of the organoids if they are larger than 500 nm (Kelava and Lancaster, 2016).

Also, most of these models have focused on the different neuronal populations even though the *in vivo* brain also consists of several other cell types such as astrocytes and oligodendrocytes.

We developed a novel *in vitro* iPSC-derived human 3D brain microphysiological system (BMPS), which is comprised of mature neurons (glutamatergic, dopaminergic and GABAergic neurons) and glial cells (astrocytes and oligodendrocytes). Quantification of the different cell types exhibited high reproducibility between experiments. Neuron and glial functions such as spontaneous electrical activity and axon myelination could be detected.

Especially the high ratio of myelination of axons in this BMPS (up to 40%) is significant, as only a few models have displayed this process *in vitro* previously. Myelin pathology is a rather frequent condition in demyelinating and inflammatory disorders such as multiple sclerosis and post-infectious diseases as well as other neurological diseases, such as acute and post-traumatic brain injury, stroke and neurodegenerative disorders (Fumagalli et al., 2016; Tse and Herrup, 2016). Moreover, the myelination process can be perturbed by exposure to chemicals and drugs (Garcia et al., 2005; Brubaker et al., 2009; Creeley et al., 2013) during brain development and in adulthood. Therefore, it is essential to develop new human *in vitro* models that also can capture the function of glial cells and allow the quantification of myelin and the study of its interaction with other CNS cell types in a reproducible way.

## 2 Materials and methods

### iPSC generation

CCD1079Sk (ATCC^®^ CRL2097™), IPS IMR90 (WiCELL) and ATCCDYP0730 human (IPS) cells (ATCC^®^ ACS1003™) fibroblasts were originally purchased from ATCC. Cell lines were karyotyped and confirmed to be human, CLR-2097 showed a short deletion in chromosome 46 after reprograming. The cell line tested negative for mycoplasma contamination. Human iPS cells were generated with EBV-based vectors as previously described (Wen et al., 2014). All studies followed Institutional Review Board protocols approved by the Johns Hopkins University School of Medicine. Colonies of iPSCs were manually picked after 3–6 weeks for further expansion and characterization. iPSCs (passage ≤ 20) were cultured on irradiated mouse embryonic fibroblasts (MEFs) in human embryonic stem cell (hESC) medium comprising D-MEM/F12 (Invitrogen), 20% KnockOut™ Serum Replacement (KSR, Invitrogen), 2 mM L-gluta- mine (Invitrogen), 100 μM MEM NEAA (Invitrogen), 100 μM β-mercaptoethanol (Invitrogen), and 10 ng/ml human basic FGF (bFGF, PeproTech). Mouse embryonic fibroblasts (MEFs) were isolated from E13 CF1 mouse embryos. Briefly, each embryo was separated from its amnion and the heads and internal organs were removed. The embryos were cut into smaller pieces using a razor blade and the remaining tissue was dissociated by incubating in trypsin/EDTA solution for 15 min. Tissue pieces were triturated using a P1000 pipette every 5 min to aid dissociation. The remaining cell suspension was centrifuged for 5 min at 300 g to form a pellet. The cell pellet was resuspended in MEF medium containing 20% FBS and cells were plated onto tissue treated T175 flasks. Fibroblasts were grown to confluency and expanded by passaging 2–3 times before irradiating by exposing to 4000 rads from a γ-radiation source and freezing down. One day prior to plating stem cells, irradiated fibroblast feeder cells were seeded onto 6 well plates pre-treated with 0.1% gelatin for 2 h. Medium was changed daily and iPSC lines were passaged using collagenase (Invitrogen, 1 mg/ml in D-MEM/F12 for 1 h at 37°C). Experiments shown in the main manuscript used ccd- 1079Sk iPSC derived cells; additional experiments with IPS IMR90 and DYP0730 are shown in Fig. S1^1^ .

### Neuronal progenitor cell (NPC) production

NPC generation followed a previously published protocol (Wen et al., 2014). After NPC generation, iPSC colonies were detached and NPCs were expanded in poly-L-ornithine and laminin-coated 175 mm flasks in StemPro^®^ NSC SFM (Life Technologies). Half of the medium was changed every day. Cultures were maintained at 37^°^C in an atmosphere of 5% CO_2_.

### BMPS differentiation

At 100% confluence, NPCs were detached by scraping and counted. 2×10^6^ cells per well were plated in 2 ml medium in non-treated 6 well-plates. Cells were grown in NPC medium for two days under constant gyratory shaking (88 rpm), allowing aggregation by using a MaxQ™ 2000 CO_2_ (ThermoFisher Scientific) plate shaker. Subsequently, medium was changed to differentiation medium (Neurobasal^®^ Electro Medium (Gib- co) supplemented with 5% B-27^®^ Electrophysiology (Gibco), 1% glutamax (Gibco), 0.02 μg/ml human recombinant GDNF (Gemini), 0.02 μg/ml human recombinant BDNF (Gemini)). Cultures were maintained at 37^°^C, 5% CO_2_ under constant gyratory shaking (88 rpm) for up to eight weeks. Differentiation medium was routinely changed every two days.

### Size measurement

Aggregates (n = 20) from three independent experiments were randomly selected per time point for obtaining pictures with a RETIGA EXi camera (QIMAGING) and measuring size using SPOT software 5.0 (SPOT IMAGING™).

### RNA and miRNA extraction

Total RNA was extracted from aggregates every week over 8 weeks of differentiation using TriPure (Roche) according to Chomczynski and Sacchi (1987). RNA quantity and purity was determined using a NanoDrop 2000c (Thermo Scientific). 1 μg of RNA was reverse-transcribed using the M-MLV Promega Reverse Transcriptase (Promega) according to the manufacturer’s recommendations. For miRNA reverse-transcription, 60 ng of RNA were reverse transcribed using TaqMan^®^ microRNA Reverse Transcription Kit in combination with miRNA specific stem-loop primers, which are part of TaqMan^®^ microRNA Expression Assay. Up to eight stem-loop primers were multiplexed in one reaction.

### Quantitative real-time reverse transcription polymerase chain reaction

The expression of genes was evaluated using specific TaqMan^®^ Gene Expression Assays (Life Technologies). miRNA expression was analyzed using TaqMan^®^ microRNA Expression Assay in combination with TaqMan^®^ miRNA Reverse Transcription Kit using the protocol described in (Smirnova et al., 2015). Table 1 shows a summary of the assayed genes. Real-time RT-PCR was performed using a 7500 Fast Real Time system machine (Applied Biosystems). Fold changes were calculated using the 2(-ΔΔCt) method (Livak and Schmittgen, 2001). β-actin and 18s were used as housekeeping genes for mRNA, and RNU44 for miRNA. Data are presented as mean ± SD, normalized to housekeeping genes and week 0.

### Immunocytochemistry of the BMPS

BMPS were fixed in 4% paraformaldehyde, washed three times in PBS, and then incubated for 1 h in blocking solution (5% normal goat serum (NGS) in PBS with 0.4% TritonX). BMPS were incubated at 4°C for 48 h with a combination of primary antibodies (Table. 2) in 3% NGS, 0.1% TritonX in PBS. BMPS were washed in PBS three times and incubated with secondary antibody for 1 h in PBS with 3% NGS at room temperature. Double immunostaining was visualized using the proper combination of secondary antibodies (e.g., goat anti-rabbit secondary antibody conjugated with Alexa 594 and goat anti-mouse secondary antibody conjugated with Alexa 488 (Molecular Probes). Nuclei were counterstained with DRAQ5 dye (Cell Signaling; 1:5000 in 1x PBS) or NucRed Live (Molecular Probes) for 15 min, mounted on slides with coverslips and Prolong Gold antifade reagent (Molecular Probes); negative controls were processed omitting the primary antibody. Images were taken using a Zeiss UV-LSM 510 confocal microscope. The experiments were performed in duplicate. 3D reconstruction was done using Imaris 7.6.4 software (Bitplane).

### Automated quantitation of cell types

BMPS were differentiated for 8 weeks. Randomly selected pictures from three experiments were acquired by confocal imaging and then analyzed with a custom algorithm created with the Cellomics Target Activation image-analysis software package (ThermoFisher Scientific). With this algorithm, cells in each image were identified using a fluorescence intensity threshold based on DRAQ5 stained nucleus. Identified nuclei were then used to define a “mask” and only pixels that fell under the defined mask were used for intensity measures of staining in other fluorescent channels on a cell-by-cell basis. Oligodendrocytes and astrocytes were quantified based on fluorescence intensity staining of CNPase, NOGO1 and GFAP antibodies.

### Myelination assessment and quantification

To calculate the percentage of axonal myelination, a semi-automated computer platform, termed computer-assisted evaluation of myelin formation (CEM) (Kerman et al., 2015), which uses NIH Image J built-in tools as well as Math lab processing functions, was used. The results were generated as pixel counts and percent values. The percent of myelinated axons was calculated by dividing the pixel count for myelin by the pixel count for axons after cell body removal and multiplying by 100. For each time point, at least 18 fields from at least two independent experiments were analyzed.

### Electron microscopy

BMPS aggregates were collected at 2, 4 and 8 weeks, respectively, and were fixed in 2% glutaraldehyde and 4% formaldehyde in 0.1 M sodium cacodylate buffer (EMS, Electron Microscopy Sciences), pH 7.4, with 3% sucrose and 3 mM CaCl_2_. Post-fixation was done with 2% osmium for 2 h. The BMPS aggregates were then stained en bloc with 2% uranyl acetate in distilled water for 30 min and subsequently dehydrated in graded ethanol and embedded using Embed 812 (EMS). Thin sections (70–80 nm) were cut on a Reichert Jung Ultracut E microtome and placed on formvar-coated 100 mesh copper grids. The grids were stained with uranyl acetate followed by lead citrate and the sections were examined with a Zeiss Libra 120 electron microscope.

### Flow cytometry

In order to quantify the percentage of NPCs and neurons within the aggregates, flow cytometry for NPC and neuronal markers was performed according to a previously published protocol (Smirnova et al., 2015) with some optimization steps for 3D cultures: Aggregates were washed once with PBS/1mM EDTA and trypsinized directly in the well using TrypLE Express containing 4 units/ml DNAse for 30 min at 37°C on a shaker. Pipetting the aggregates up and down with a 1 ml syringe and a 26G3/8 needle ensured generation of a single cell suspension. Cells were counted, washed once with PBS/1mM EDTA, fixed with 2% PFA for 20 min at 4°C, washed twice with PBS/1%BSA and blocked for 30 min in blocking solution (PBS/1% BSA/0.15% saponin/10% NGS). 1×10^6^ cells were stained for 1 h at 4°C with fluorochrome-conjugated antibodies dissolved in blocking solution (Table. 3).

Unstained cells as well as cells incubated with isotype controls were used as negative controls to set the gates for measurements. Cells were washed twice with PBS/1% BSA/0.15% saponin, and once with PBS/1% BSA. Flow cytometry was performed using a Becton Dickinson FACSCalibur system by measuring 10^4^ gating events per measurement. Data was analyzed using FlowJo v10 software (Table. S1^1^).

### Microelectrode array (MEA) recordings

After eight weeks of differentiation, BMPS were plated on 48-well MEA plates (Axion Biosystems) previously coated with Matrigel (BD Bioscience). Spontaneous electrical activity was recorded using the “Maestro” MEA platform and Axion’s Integraded Studio (AXIS) software (Axion Biosystems) over ten days. Each well of the 48-well MEA plate contains 16 individual microelectrodes (~40–50 μm diameter, center-to-center spacing 350 μm) with integrated ground electrodes, resulting in a total of 768 electrodes per plate. All recordings were performed at a constant temperature of 37°C. Prior to 20 min of recording, the MEA plates were placed in the Maestro MEA platform and equilibrated for 5 min. AXIS software was used to control the heating system and to monitor the recordings, which involves simultaneous sampling of the channels at 12.5 kHz/ channel with a gain of 1200x and a band pass filter of 200–5000 Hz. After recording, the RAW-files were re-recorded with AXIS to convert the data into a spike file, which includes spike timing and profile information. A variable threshold spike detector was used for the spike file and was set at six times standard deviations of the rms-noise on each channel. The spike file was later used for data analysis with NeuroExplorer^®^ (Nex Technologies) to convert data into Microsoft Excel files. Using the function rate histogram, a summary of the spikes of all electrodes of one plate was put into one Excel sheet. Only electrodes that recorded activity higher than 0.05 spikes/sec at least once over the time measured were included for data analysis.

### Statistical analysis

For myelination quantification at the different time points, a Kruskal-Wallis test was employed, statistical significance was considered for p values < 0.05.

## 3 Results

### 3.1 Development of a size-controlled BMPS

**Fig. 1:**
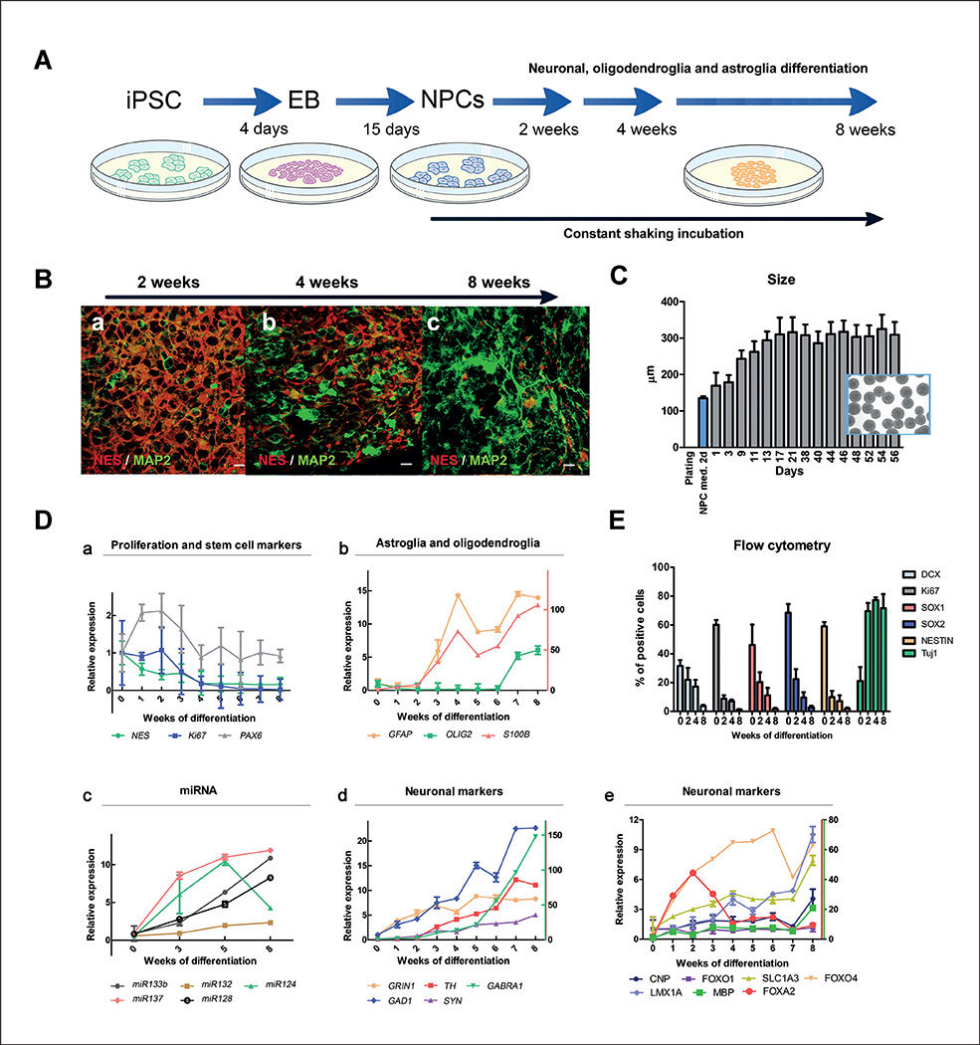
Characterization of BMPS during differentiation I (A) Diagram of the differentiation protocol. (B) Co-immunostaining of MAP2^+^ neurons with the maturation marker Nestin at 2, 4 and 8 weeks. Scale bar: 20 μm. (C) Size of aggregates measured during the 3D neuronal differentiation. Aggregates (n = 20) from three independent experiments were randomly selected per time point for obtaining pictures and measuring size using SPOT software 5.0. The blue bar (NPC med. 2d) represents the size of aggregates cultured in 3D for two days in NPC medium while from day 1 the cells were cultured in differentiation medium. Results are expressed as mean ± SD. Cells were kept for two days in NPC medium, indicated as “NPC med. 2 d”. Inserted phase contrast image shows several aggregates at 13 days of differentiation with an average diameter of 310 μm. (D) BMPS mRNA and miRNA expression of different markers during differentiation (b: right y-axis relative quantification of *S100B* and d: right y-axis relative quantification of *GABRA1, TH; e: FOXA2, FOXO4* and *MBP).* (E) Flow cytometry population analysis of BMPS at different stages of differentiation.

**Fig. 2:**
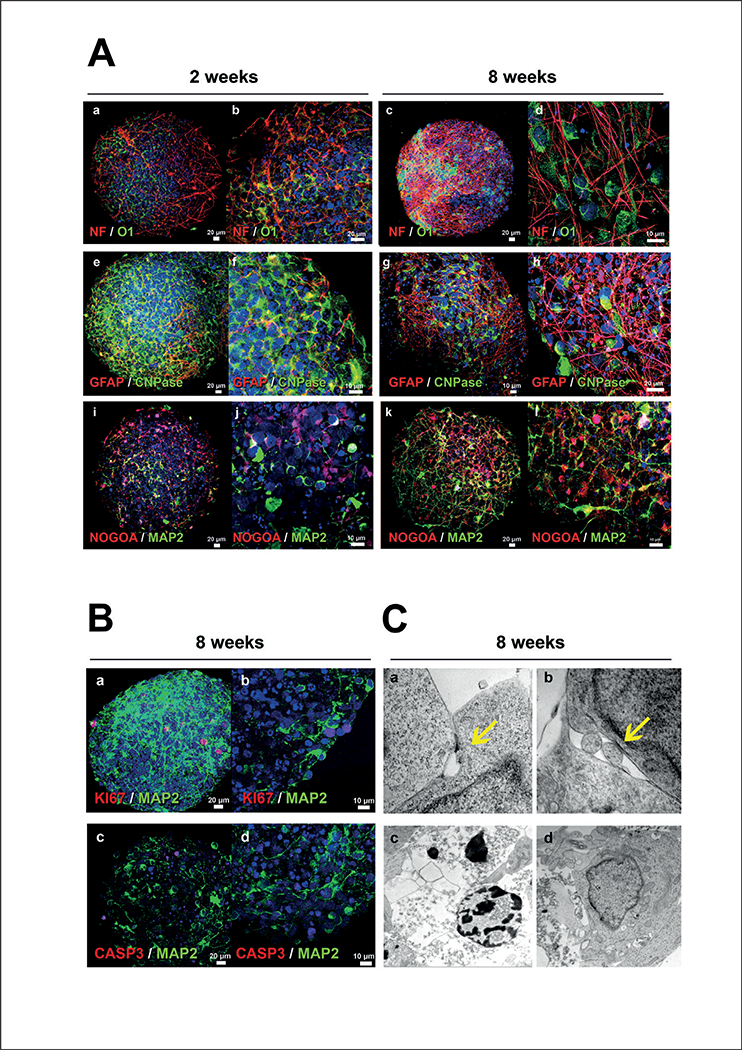
Characterization of BMPS during differentiation II (A) Comparison of expression of neuronal and glial markers at 2 and 8 weeks. At 2 weeks, oligodendrocytes (O1, CNPase and NOGOA) were identified without a preferential localization (a, b, e, i, j), later they resemble human oligodendrocytes and localize in close proximity with axons (c, d, h, k, l). At 2 weeks, there are few MAP2^+^ cells and they lack an identifiable neuronal shape (i,j) whereas at 8 weeks the MAP2^+^ cells acquire a well-defined dendritic network (k, l). The number of astrocytes (GFAP^+^) and density of the astroglial network increased with time of differentiation (e, f vs. g, h). (B) Co-immunostaining of neurons (MAP2) with cell-division marker Ki67 showed that some cells are still dividing at 8 weeks (a, b). There was also a small degree of apoptosis demonstrated by positive staining with CASP3 at 8 weeks (c). CASP 3-positive nuclei did not co-localize with mature neurons (d). (C) Ultrastructure analysis by electron microscopy at 8 weeks showed evidence of cell-to-cell junctions demonstrating functional interactions between the cells (arrows, a, b). Nuclear variation was confirmed by the presence of a few apoptotic nuclei (c) compared to normal healthy nuclei (d). NF, Neurofilament-heavy-chain; MAP2, microtubule-associated-protein 2; GFAP, glial-fibrillary-acidic protein; O1, Olig1 ; CNPase, 2’,3’-cyclic- nucleotide-3’-phosphodiesterase; CASP3, caspase-3.

Different technologies have been used to generate BMPS over the last three years, however, most studies published to date do not allow control of the organoid size and shape. The BMPS model established here followed a stepwise differentiation protocol (Fig. 1A). In the final step, cells were differentiated into various neuronal and glial cell types during constant gyratory shaking. This technique induced spherical shapes and controlled the size (< 350 μm) of the BMPS (Fig. 1C), i.e., a size that avoids necrosis at the center of the sphere from occurring due to nutrient and oxygen deprivation observed in larger spheroids (Fig. 2B c-d). This technique allows the production of several batches under different chosen conditions at the same time. Without the shaking, aggregates tend to stick together, grow in different shapes, attach to the bottom and at some point become necrotic in the middle of the sphere (data not shown).

Two days after initiation of aggregation in NPC medium, spheres were on average 130 ± 5 μm in diameter (Fig. 1C). From day 17 onwards, the diameter remained constant around 310 μm.

Different cell lines behave differently, and optimization of the shaking speed is required in order to control the shape (Fig. S1^1^).

### 3.2 Expression of CNS-specific genes and miRNA during development and differentiation of the BMPS model

In order to characterize different stages of the differentiation and the maturation process, BMPS were collected every week over 8 weeks of differentiation. Analysis of different neuronal and glial cell-specific genes by quantitative RT-PCR was performed to characterize the presence of neurons, astrocytes, oligodendrocytes and NPCs (Fig. 1D, Fig. S1A^1^).

Gene expression of the cell proliferation marker *Ki67* remained similar to NPC until 2 weeks of differentiation (Fig. 1D a) when the expression started to decrease. The remaining *Ki67* expression is likely due to the presence of a small population of NPCs and other proliferating cell types such as oligodendrocytes and astrocytes (Fig. 1D b).

Astrocyte specific genes *(S100B* and *GFAP)* showed a constant increase in expression after 2 weeks, while oligodendrocyte genes were observed later, after 6 weeks of differentiation, as shown by *OLIG2* gene expression (Fig. 1D b).

MicroRNAs (miRNA), known as posttranscriptional regulators of developmental timing, have been established as markers of the neural differentiation process (Li and Jin, 2010) and were used here to characterize the BMPS (Fig. 1D c). *mir-124,* the most abundant brain miRNA, was strongly expressed in the earlier stages of differentiation, then somewhat down-regulated at 8 weeks of differentiation. This finding correlates with previous studies, where *mir-124* was shown to promote neuronal lineage commitment at early stages of neural stem cell specification by targeting anti-neuronal factors (Li and Jin, 2010). mir-128, a modulator of late neural differentiation, was strongly up-regulated after 5 weeks of differentiation. *mir-137,* the most induced miRNA over time in our system, is known as a regulator of neural differentiation in embryonic stem cells (ESCs) (Tarantino et al., 2010). *mir-132* and *mir-133b,* which are involved in regulation of dopaminergic neuron maturation and function, were induced at week 3 of differentiation. These results support the view of a coordinated mechanism of neuronal differentiation as reflected by the patterns of neuronal gene and miRNA expression and neuronal neurotransmitter identity.

Gene expression of specific neurotransmitters or their receptors was used to characterize the identity of different neuronal populations (Fig. 1D d). *GRIN1,* which encodes the essential glutamate (NMDA) receptor subunit ζ−1 (Monyer et al., 1992), was increased at very early stages of differentiation (one week after induction of differentiation) and continued to increase up to 5 weeks (Fig. 1D d). Similarly, glutamate decarboxylase 1 (GAD1), a GABAergic neuronal gene marker, showed a constant increment of expression during the 8 weeks of differentiation. *GABRA1,* which encodes the γ-aminobutyric acid *(GABA)* receptor showed a steady increase of expression after 2 weeks and reached its highest level at 8 weeks (150-fold the value at week 0) (Fig. 1D d). The expression of synapsin (SYN), a specific marker for synapses, was constantly increasing over the time starting from 3 weeks of differentiation (Fig. 1D d). The expression of tyrosine hydroxylase (*TH*), a gene that identifies dopaminergic neurons, was observed first after 3 weeks, showing delayed differentiation compared to glutamatergic neurons, a finding, that correlates with the expression pattern of *mir-132* and *mir-133b* (Fig. 1D c).

Moreover, other markers for specific parts of the brain, such as ventral midbrain neuron markers LMX1A, FOXO1 and FOXA2 (Hedlund et al., 2016; Stott et al., 2013), cerebral cortex marker FOXO4, markers for L-myelination CNP and MBP (Li and Richardson, 2008; Agrawal et al., 1994), and a marker for L-glutamate transport SLC1A6 (Sery et al., 2015) were studied (Fig. 1D e).

### 3.3 Characterization of marker expression by flow cytometry shows neuronal maturation in the human iPSC derived BMPS

**Fig. 3:**
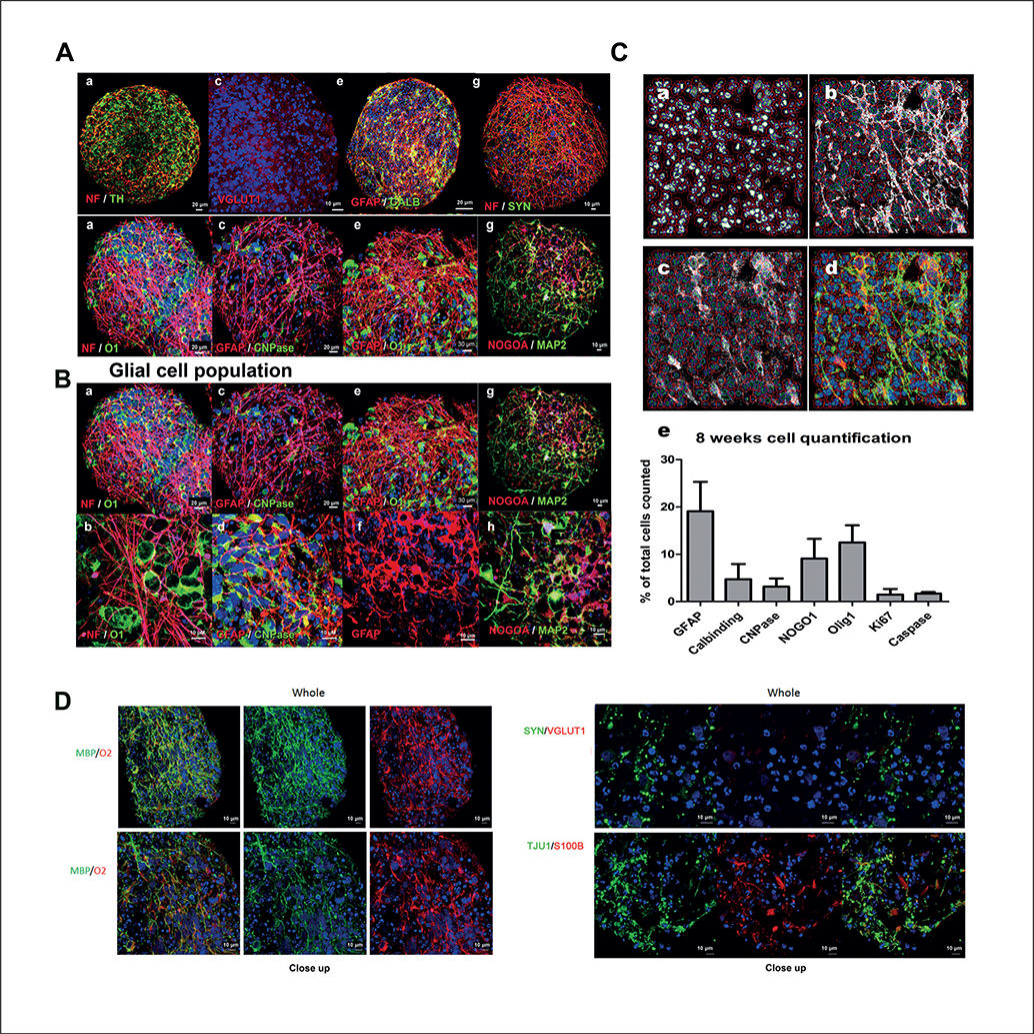
Morphologic characterization of mature human BMPS (A) At 8 weeks, neuronal populations exhibited a diversity of neurotransmitter identities as shown by identification of dopaminergic TH^+^ (a, b), glutamatergic VGLUT1^+^ (c, d) and GABAergic calbindin^+^ (e, f) neurons. Neurons disclosed characteristic axons (NF) and synapsins (SYN) (g, h). (B) Two distinctive glial populations were identified in close interaction with neuronal populations, GFAP^+^ astroglia, and CNPase^+^, O1^+^, NOGOA^+^ oligodendroglia. O1^+^ oligodendrocytes were closely associated with axonal processes (NF) (a, b), CNPase^+^ oligodendroglia appeared mixed among GFAP^+^ astroglia (c, d) and exhibited the characteristic multipolar glial processes, which extended from the perykaria (e, f). NOGOA^+^ cells were associated with MAP^+^ neurons (g, h). (C) Example of custom algorithm created using the Cellomics Target Activation image-analysis software package to study astrocytes and oligodendrocytes (a, b, c, d). Quantification of cell populations as a percentage of the total nuclei count of GFAP, Calbindin, CNPase, NOGOA, Olig1, Ki67 and Caspase positive cells at 8 weeks (e). Randomly selected pictures from three experiments were acquired. The algorithm associated an astrocyte or oligodendrocyte cell body with a nucleus and quantified with respect to the total nucleus count (D) Co-expression of mature oligodendroglia markers (MBP and O2). (E) Expression of neuronal markers (VGLUT, TUJ1, SYN).

In order to quantify cell populations in the iPSC-derived BMPS and verify reproducibility between experiments and batches of the cell line (CCD1079Sk, CRL-2097), marker expression was investigated by flow cytometry using CNS-specific antibodies at different stages of differentiation (Table. 1). 60% of cells displayed the proliferation marker Ki67 at the NPC stage (week 0), which was reduced during differentiation down to 9% at 2 weeks, 7% at 4 weeks and 1% at 8 weeks (Fig. 1E). Automated quantitation showed similar numbers of Ki67 positive cells (Fig. 3C e). This confirms the gene expression data and indicates a fast drop in the number of proliferating cells after induction of differentiation.

The number of SOX1-, SOX2- and NES-positive (NPC marker) cells in the NPC population (week 0) was 46%, 68% and 60%, respectively. SOX1, SOX2 and NES expression was also reduced dramatically with differentiation, showing very small positive populations at 8 weeks (2%, 3% and 2%, respectively) (Fig. 1E). The change in the cell population during differentiation was corroborated by the measurement of doublecortin (DCX), a microtubule-associated protein expressed in neuroblasts and immature neurons: The number of DCX-positive cells in NPC (week 0) was around 30%, which dropped to 22% at 2, 17% at 4 and 4% at 8 weeks, respectively.

The marker for mature neurons, Tuj1 (neuron-specific class III β-tubulin) presented the opposite pattern. Low levels of TUJ1-positive cells at the NPC stage (week 0) increased to up to 70% of positive cells after 2 weeks of differentiation that remained constant until 8 weeks.

Quantification of the cell populations in at least three independent experiments showed low variability between cultures, demonstrating the reproducibility of the system. The variation (SD) between experiments decreased with the cell differentiation process and was very small at the latest maturation stage (8 weeks); DCX SD 0.9%, Ki67 SD 0.2%, SOX1 SD 0.7%, SOX2 SD 1.2%, NES SD 0.7% and Tuj1 SD 9,8% (Fig. 1E). These results indicate that after 8 weeks of differentiation the cellular composition is similar and shows high reproducibility between different BMPS experiments.

### 3.4 Immunohistochemistry and electron microscopy reveal a variety of differentiated and mature cell types in the BMPS, including functional oligodendrocytes

In order to further assess the cellular composition and the maturation of the cells within the human BMPS, immunohis- tochemistry and electron microscopy techniques were employed. We observed different neuronal subtypes in the BMPS including dopaminergic (TH-positive neurons), glutamatergic (VGLUT1 -positive neurons) and GABAergic interneurons (Calbindin-positive neurons) (Fig. 3A and E). Moreover, the BMPS matured during the differentiation process as seen by decreased Nestin-positive cells (Fig. 1B) and increased cell-cell interactions (neuron-neuron and neuron-glia) as subsets of neurons showed several processes resembling dendritic (MAP-2) and axonal (NF) projections (Fig. 1B, 2A a-d, i-l, 2B, 3A a-b, g-h, and 3B a-b, g-h, Supplementary video^2^ ). In addition, cell-to-cell junctions could be observed by electron microscopy imaging indicating functional interactions between the cells (Fig. 2C a,b). The neuron-neuron interactions were further demonstrated with immunohistochemistry for synapsin (SYN), a marker of the major synaptic vesicle protein (Fig. 3A g,h).

**Fig. 4:**
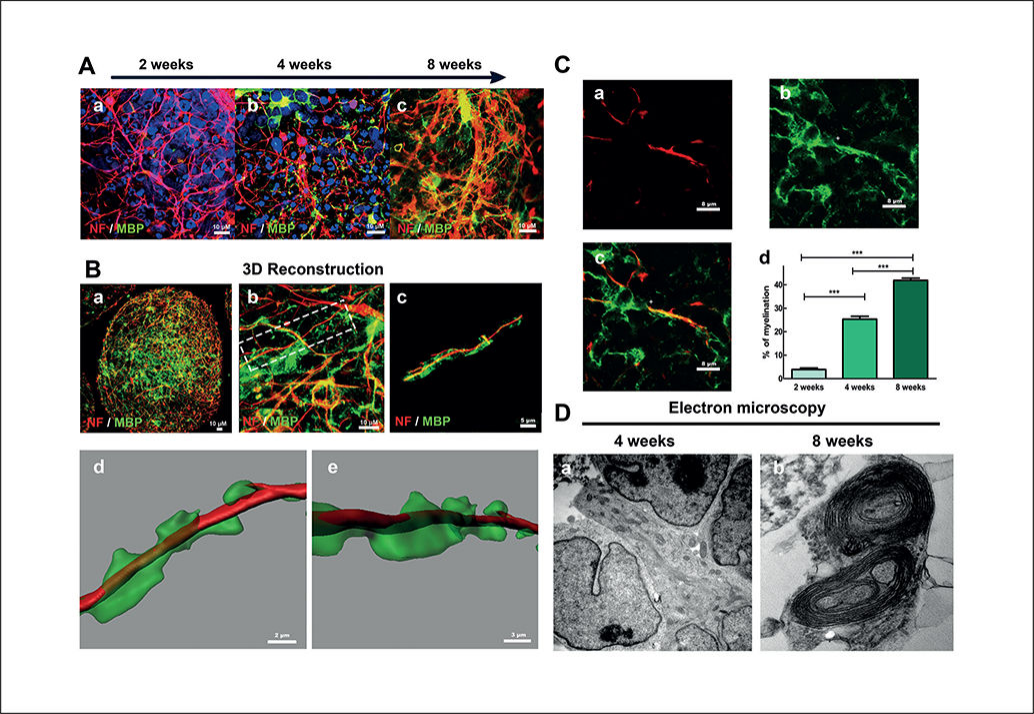
Myelination in BMPS (A) Co-immunostaining of neurons (NF) and the myelin marker MBP at 2, 4 and 8 weeks of differentiation (a, b, c, respectively) showed progressive increase of MBP^+^ cells in association with axonal processes. (B) 3D-reconstruction based on confocal z-stacks at 8 weeks demonstrating a “wrapping” myelinating process, which resembles the myelination of axons in human CNS. (C) MBP^+^ oligodendrocytes (green) issued processes in close association with axons (red) and seemed to enwrap them at 8 weeks (a, b, c). Myelination calculated as the mean percentage MBP positive oligodendrocyte processes coverage of NF-positive axons (a, b, c) at 2, 4 and 8 weeks in at least 18 microscopy fields from at least 3 individual BMPS in 2 independent experiments showed significant increase of myelination observed with time of differentiation (d) (***, p < 0.001; Kruskal-Wallis test). (D) Electron microscopy analysis of BMPS at 4 (a) and 8 (b) weeks of differentiation identified morphology of axonal structures and cells, which appeared to be oligodendrocytes. Myelinating-like processes, which closely resembled cross-sections of myelinated axons of the CNS, were identified at 8 weeks of differentiation.

A subset of neuroglial cells exhibited immunoreactivity for markers such as NOGOA, O1, O2 and CNPase (Fig. 2A, Fig. 3B, 3D and Fig. S1A^1^), which indicates the presence of mature oligodendrocytes in the BMPS (Deng and Poretz, 2003; Schwab, 2010). Automatic image quantification showed that oligodendrocytes (CNPase, NOGOA and Olig1) comprised 3, 9 and 11% of the total cell population, respectively, at 8 weeks of differentiation (Fig. 3C e) in three different experiments. Similar to the *in vivo* physiology, these cells were immunoreactive for myelin basic protein (MBP) (Fig. 3D and4), which characterizes myelinating oligodendrocytes (Fancy et al., 2011). Moreover, they had morphological features of normal human oligodendrocytes *in vivo* and appeared in close contact with neuronal processes (Fig. 2A, Fig. 3B and Fig. 4). In addition, electron microscopy showed the myelin wrapping around the axons (Fig. 4D).

This pattern of immunostaining suggests that oligodendrocytes within the BMPS are functional and myelinate axons. MBP expression increased with time of differentiation (Fig. 4A). Morphometric studies of neuronal processes identified by immunostaining with NF antibodies and MBP markers were used to estimate the percentage of myelinated axons within the BMPS (Kerman et al., 2015) with an average of 4% ± 2.78% at 2 weeks, 25% ± 7.9% at 4 weeks and 42% ± 6.8% at 8 weeks of differentiation (p < 0.001) (Fig. 4C). All analyzed BMPS showed a similar extent of myelination at the same differentiation window. Ultrastructural analysis by electron microscopy demonstrated oligodendrocyte cell projections that enwrapped cell processes resembling axons after 8 weeks of differentiation (Fig. 4D b). In addition, 3D reconstruction was done using Imaris 7.6.4 software. For this analysis, a z-stack of confocal imagines is used. The reconstruction (Fig. 4B) confirms the wrapping of the axon by myelin found in the electron microscopy experiments.

GFAP-positive cells formed numerous cell processes organized in a network typical for human astrocytic processes *in vivo*, with established contacts to other glial cells and neurons (Fig. 2A e-h, 3A c-f and 3B c-f). Imaging quantification revealed 19% GFAP-positive astrocytes in the total population (Fig. 3C e).

The morphology of cell nuclei observed by immunocyto- chemistry and electron microscopy showed a few variations attributed to (i) cell proliferation as seen by positive staining for Ki67 and NES markers and (ii) nuclear fragmentation likely associated with apoptosis as indicated by Caspase-3 staining (Fig. 2B, Fig. 2C c,d). This variation likely reflects the active stages of cell differentiation that BMPS exhibit during development (Meijer et al., 2012). Importantly, Caspase-3^+^ nuclei did not concentrate in the center of the spheres (Fig. 2B) and thus do not appear linked to deprivation of oxygen or nutrients. Caspase-3 was quantified at 8 weeks in BMPS showing 1.68% ± 0.3% caspase-3 positive cells (Fig. 3C e).

### 3.5 The BMPS model exhibits neuronal functionality revealed by spontaneous electrical activity

**Fig. 5:**
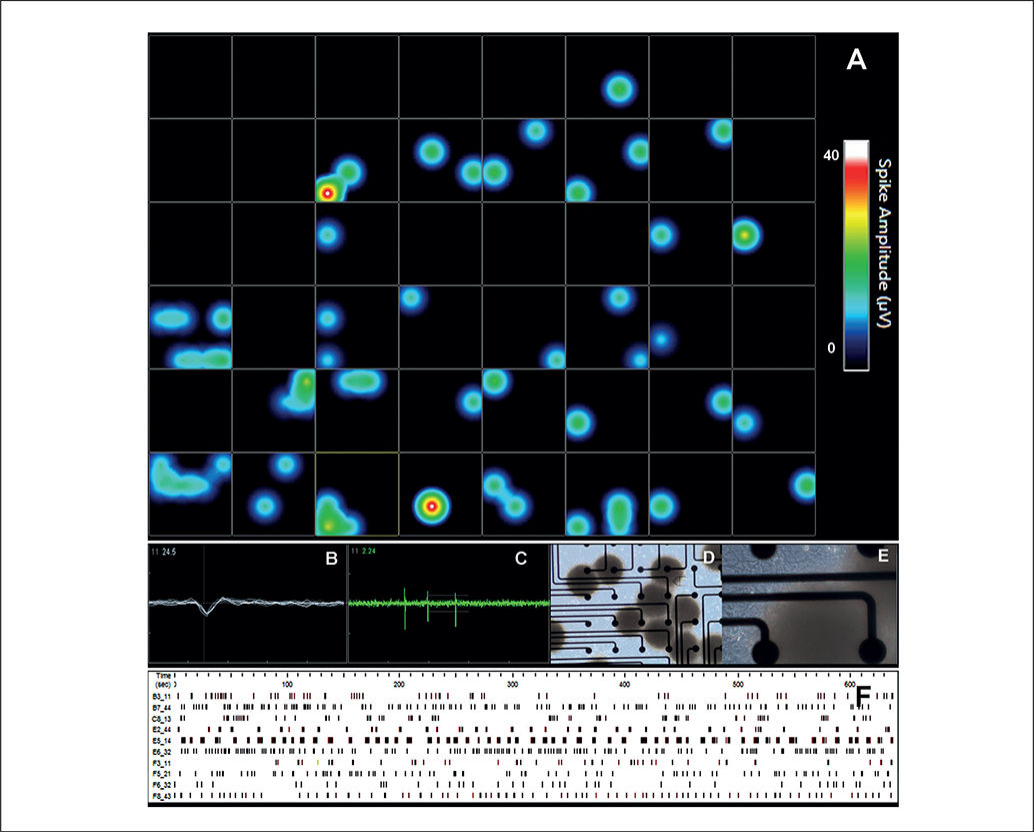
Electrical activity as a neuronal function of the BMPS Cells were cultured in 3D for 8 weeks and then cultured in 48-well MEA plates for 2 more weeks. (A) Heat map recordings from a 48-well plate. Illustration of an active well showing (B) spike morphology and (C) spike activity. (D and E) Phase-contrast imaging of the BMPS on MEAs, electrode diameter is 40–50 μm and inter-electrode space is 350 μm. (F) Activity pattern recordings over 0.05 spikes/sec of the electrode over 10 min.

To test the neuronal physiological properties of the cells within the BMPS model, spontaneous electrical activity in BMPS was analyzed by multi-electrode array (MEA) (Fig. 5D). BMPS at 6 weeks of differentiation were plated on 48-well MEA plates previously coated with Matrigel. Spontaneous electrical activity was measured from one week after plating up to two weeks. The activity was recorded for 20 minutes on seven different days. Maximum activity was observed from day 5 to day 7. Electrodes were considered active when the recorded activity was above 0.05 spikes/sec. Figure 5A shows a representative heat-map of a 48-well MEA plate from one 20-minute recording (day 6 of recording period). The heat map represents the spike amplitude (μΥ) with a minimum of 0 μV and maximum of 40 μV (Fig. 5A). The spikes showed a common waveform between different electrodes and measurements (Fig. 5B) and neurons were firing repeatedly (Fig. 5C). Data was derived from 25 electrodes, distributed over wells. 20 to 40% of these 25 electrodes reached the threshold of 0.05 spikes/sec during each recording. Figure 5F shows the spike events of active electrodes from one representative 20-minute recording. These data show the potential use of MEA for measuring electrical activity of the 3D BMPS. However, further optimization of the protocol is needed.

## 4 Discussion

Stem cell-derived brain model systems developed in the past few years have shown the ability to recapitulate some of the *in vivo* biological processes (Juraver-Geslin and Durand, 2015; Nakano et al., 2012; Krug et al., 2014) and have an advantage over other classical *in vitro* models as they facilitate the study of various differentiation mechanisms, developmental processes and diseases (Lancaster et al., 2013). Combining stem cells with emerging culturing techniques such as 3D culture (Alépée et al., 2014; Hartung, 2014) promotes the development of new, more complex human *in vitro* models, such as microphysiological systems (Andersen et al., 2014; Marx et al., 2016), that enhance modeling of the *in vivo* brain (Pasca et al., 2015; Lancaster et al., 2013). However, they often require complicated protocols that may reduce the reproducibility of the system and make it difficult to use in other fields such as chemical toxicity and drug screening. Some of these complex organoid models are also limited by large diameters, which can lead to extensive cell death in the middle due to insufficient diffusion of oxygen and nutrients (Lancaster et al., 2013) and other artifacts. Recently, attempts to generate more reproducible brain microphysiological systems were published using rat primary cortical tissue (Dingle et al., 2015).

In this study, we have developed a human *in vitro* model using a gyratory shaking technique that enables reliabe generation of a high number (about 500 per six-well plate) of viable BMPS that are homogeneous in size and shape. Control of size allowed us to keep cell aggregates below 350 μM in diameter and avoid disparate morphology and/or necrosis in the center of the spheres. Moreover, the BMPS showed reproducible cell composition by immunomorphological quantification (Fig. 3C e), assessment of imaging-based endpoints (Fig. 4E) and flow cytometry analysis (Fig. 1E). Results shows a composition of 20% GFAP positive astrocytes and 70% TUJ1 positive mature neurons at 8 weeks (Fig. 3C e), which is in agreement with existing data published by Pasca et al. (2015).

The 3D differentiation protocol for the BMPS covers stages from neuronal precursors to different cell types of the mature CNS. Gene expression studies, flow cytometry, image analysis, immunostaining and miRNA studies show an increase of cell maturation markers, which follow the BMPS differentiation. At two weeks, BMPS consisted of an immature population of cells, showing minimal neuronal networks, a low percentage of mature astrocytes and oligodendrocytes, and minimal but early stages of myelin basic protein (MBP) expression (Fig. 1D, Fig. 2A). iPSC differentiation into mature BMPS was indicated by decreasing NES expression over time and a progressive expression of mature neuronal and glial markers such as MAP2, GFAP, O1 and MBP. At 2 weeks we observed the presence of GABAergic neurons (Fig. 1D d), dopaminergic neurons (TH marker, Fig. 1D d and 1A a) and glutamatergic neurons (Fig. 3A c), documented by immunohistochemistry and real-time PCR data (Fig. 1D, Fig. 3A,E). Moreover, the BMPS showed spontaneous electrical activity (Fig. 5), indicating neuronal functionality of the system.

Most of the recent BMPS published are entirely focused on neurons and not glial populations (Park et al., 2015; Dolle et al., 2014). Since astrocytes and oligodendrocytes play important roles during neuronal development, plasticity and injury, the presence of glial cell populations in this BMPS model provides an excellent opportunity for the evaluation of neuronal-glial interactions and the role of glia in pathogenesis and toxicity processes. Astrocytes have an important role in protecting neurons, increasing neuronal viability and mitochondrial biogenesis from both exogenous (e.g., chemicals) and endogenous toxicity (Shinozaki et al., 2014; Aguirre-Rueda et al., 2015), especially against oxidative stress (Shao et al., 1997; Schwab and McGeer, 2008). Thus, their presence in a biological system designed to study disease and neurotoxicity is crucial. Immunohistochemistry and RT-PCR results showed increasing numbers of astrocytes (GFAP^+^ cells) in the BMPS model (Fig. 2A, Fig. 1D, Fig. 3B) reaching 19% astrocytes of the total cell population at 8 weeks (Fig. 3C). This is earlier than in previously described cortical spheroids, where similar proportions of GFAP^+^ cells were observed first at day 181, at day 86 the number of GFAP^+^ cells was below 10% (Pasca et al., 2015). We acknowledge that there is a subset of the GFAP^+^ cells that could still be radial glia as there is some nestin expression even after 8 weeks of development (Lyck et al., 2008; DeAzevedo et al., 2003).

The most novel element of this BMPS is the presence of mature human oligodendrocytes with myelination properties. Immunocytochemical and ultrastructural studies confirmed the morphological identity of these cells (Fig. 3B and4) as multiple markers for mature oligodendrocytes were expressed by rounded cells with branching processes and membrane sheaths that are similar to the ones found in humans *in vivo.* The structure and morphology was further confirmed by electron microscopy images. Quantitative assessment of the myelination process of MBP immunostaining along axons showed an increase over time of differentiation reaching 42% of myelinated axons at 8 weeks (Fig. 4C). 3D reconstruction of confocal z-stacks images (Fig. 4B) and electron microscopy confirmed the wrapping of axonal structures after eight weeks of differentiation (Fig. 4D). These findings are of particular relevance since myelin is a critical element for proper neuronal function and development, and the covering of axons by myelin allows faster action potential transmission, reduces axonal energy consumption and protects the axons from degeneration (Nave, 2010). Furthermore, recent evidence suggests that oligodendrocytes and myelin have a role in the metabolic support of axons independent of their role in action potential conduction, highlighting their importance in neuronal survival (Saab et al., 2013). Very few *in vitro* models have been developed that can capture the myelination process and most of them are derived from primary rodent cells (Lari- osa-Willingham et al., 2016; Schott et al., 2016; Bourre et al., 1979). Some recent studies demonstrate human myelinating oligodendrocytes derived from iPSC (Uemura et al., 2014; Wang et al., 2013), however, only by introducing the cells into mice *in vivo.* Altogether, cell morphology, immunostaining and cell-cell interactions shown by neuronal and glial cell populations demonstrate that the BMPS recapitulates cellular types and patterns of interactions seen in the human CNS and can therefore be considered organotypic.

To our knowledge, this is the first time that a 3D human microphysiological system, consisting of different types of neurons and glial cells, has achieved such a high percentage of myelination. The ability to assess oligodendroglia function and mechanisms associated with myelination in this BMPS model provides an excellent tool for future studies of neurological disorders such as multiple sclerosis and other demyelinating disorders. As an illustration it was recently discovered that astroglia could promote oligodendrogenesis via secreted molecules (Jiang et al., 2016). A human BMPS that consists of neurons, astrocytes and oligodendrocytes is essential to evaluate this mechanism further and to develop a potential therapy for demyelinating disorders.

In conclusion, the BMPS showed here replicates crucial aspects of brain physiology and functionality. The potential for studying developmental and neurodegenerative disorders, brain infections, toxicity and trauma with such a system is growing. Furthermore, the potential to use iPSCs from different donors adds a personalized component to these studies. The high re-producibility and relatively simple protocol enable future medium-throughput (96-well format) testing of chemicals, drugs and their potential to induce or treat diseases.

## Supplementary Material

Supp

## Figures and Tables

**Table 1:** Primers used to determine gene and miRNA expression

Assay ID	Assay type	Catalog number	Assay name
Hs01060665	TaqMan^®^ Gene Expression Assay	4331182	BACT
Hs99999901	TaqMan^®^ Gene Expression Assay	4331182	18S
Hs04187831	TaqMan^®^ Gene Expression Assay	4331182	NES
Hs01032443	TaqMan^®^ Gene Expression Assay	4331182	Ki67
Hs01088112	TaqMan^®^ Gene Expression Assay	4331182	PAX6
Hs00909233	TaqMan^®^ Gene Expression Assay	4331182	GFAP
Hs00300164	TaqMan^®^ Gene Expression Assay	4331182	OLIG2
Hs00902901	TaqMan^®^ Gene Expression Assay	4331182	S100B
Hs00609557	TaqMan^®^ Gene Expression Assay	4331182	GRIN1
Hs00165941	TaqMan^®^ Gene Expression Assay	4331182	TH
Hs00971228	TaqMan^®^ Gene Expression Assay	4331182	GABRA1
Hs01065893	TaqMan^®^ Gene Expression Assay	4331182	GAD1
Hs00199577	TaqMan^®^ Gene Expression Assay	4331182	SYN1
Hs00232429	TaqMan^®^ Gene Expression Assay	4331182	TBR1
Hs01003383	TaqMan^®^ Gene Expression Assay	4331182	SNCA
Hs01003430	TaqMan^®^ Gene Expression Assay	4331182	KEAP1
Hs00929425	TaqMan^®^ Gene Expression Assay	4331182	NDUFB1
Hs01101219	TaqMan^®^ Gene Expression Assay	4331182	ATP5C1
Hs00919163	TaqMan^®^ Gene Expression Assay	4331182	ATP50
Hs00354836	TaqMan^®^ Gene Expression Assay	4331182	CASP1
Hs00263981	TaqMan^®^ Gene Expression Assay	4331182	CNP
Hs01054576	TaqMan^®^ Gene Expression Assay	4331182	FOXO1
Hs00188193	TaqMan^®^ Gene Expression Assay	4331182	SLC1A3
Hs00936217	TaqMan^®^ Gene Expression Assay	4331182	FOXO4
Hs00892663	TaqMan^®^ Gene Expression Assay	4331182	LMX1A
Hs00232764	TaqMan^®^ Gene Expression Assay	4331182	FOXA2
1182	TaqMan^®^ microRNA Assay	4427975	mmu-miR-124a
2216	TaqMan^®^ microRNA Assay	4427975	hsa-miR-128a
457	TaqMan^®^ microRNA Assay	4427975	hsa-miR-132
2247	TaqMan^®^ microRNA Assay	4427975	hsa-miR-133b
1129	TaqMan^®^ microRNA Assay	4427975	mmu-miR-137
1094	Control miRNA Assay	4427975	RNU44

**Table 2:** Primary antibodies used in the immunocytochemical analysis

Antibody	Host	Type	Source	Dilution
NF-H	Rabbit	Polyclonal	Enzo	1:1000
GFAP	Rabbit	Polyclonal	Dako	1:500
Olig1	Mouse	Monoclonal	Millipore	1:500
CNPase	Mouse	Monoclonal	Millipore	1:500
Calbindin	Mouse	Monoclonal	SIGMA	1:500
NOGO-A	Rabbit	Polyclonal	Santa Cruz	1:500
Map2	Mouse	Monoclonal	Chemicon	1:1000
MBP/ SMI99	Mouse	Monoclonal	COVANCE	1:1000
SMI-32	Mouse	Monoclonal	Stenberger Monoclonals	1:2000
Synaptophysin	Mouse	Monoclonal	SIGMA	1:500
VGLUT1	Rabbit	Polyclonal	Alpha Diagnostic	1:500
TH	Mouse	Monoclonal	Millipore	1:250
Nestin	Rabbit	Polyclonal	Millipore	1:200
Ki67	Rabbit	Polyclonal	abcam	1:100
Caspase3	Rabbit	Polyclonal	R&D	0.2 μg/ml
OLIG1	Mouse	Monoclonal	Millipore	1:200
TUJ1	Mouse	Monoclonal	Stemcell technologies	1:200
S100B	Rabbit	Polyclonal	Santa Cruz	1:200

**Table 3:** Antibodies used for flow cytometry analysis

Antibodies	Host	Type	Source	Dilution
Alexa Fluor^®^ 647 Nestin	Mouse	Monoclonal, clone 25	BD Pharmingen	1:05
Alexa Fluor^®^ 488 -III-Tubulin	Mouse	Monoclonal, clone TUJ1	BD Pharmingen	1:05
PerCP-Cy™ 5.5 Sox2	Mouse	Monoclonal, clone 030–678	BD Pharmingen	1:20
PerCP-Cy™ 5.5 Sox1	Mouse	Monoclonal, clone N23–844	BD Pharmingen	1:20
PE Doublecortin	Mouse	Monoclonal, clone 30	BD Pharmingen	1:20
Alexa Fluor^®^ 647 Ki67	Mouse	Monoclonal, clone B56	BD Pharmingen	1:20
