# Supplementary material for: A Human Brain Microphysiological System Derived from Induced Pluripotent Stem Cells to Study Neurological Diseases and Toxicity
Source: ALTEX. Author manuscript; Available in PMC 2018 Jul 16. (PMC6047513; doi:10.14573/altex.1609122)
Supplement: Supp [file NIHMS977950-supplement-Supp.pdf]

Pamies et al.:

A Human Brain Microphysiological System Derived from Induced Pluripotent Stem Cells to Study Neurological Diseases and Toxicity

Supplementary Data

Tab. S1: Flow cytometry raw data

**NPCs**			
Antibody name	n	Mean %	SEM
DCX	4	3.96	2.00
Ki67	4	2.27	6.79
SOX1	4	60.07	3.30
SOX2	4	46.02	14.20
Nestin	4	68.42	6.03
Tuj1	4	58.96	3.05
**2 weeks**			
Antibody name	n	Mean %	SEM
DCX	4	21.89	8.24
Ki67	3	8.85	3.18
SOX1	3	20.27	6.86
SOX2	3	22.31	6.99
Nestin	4	9.87	4.36
Tuj1	3	69.52	5.61

**4 weeks**			
Antibody name	n	Mean %	SEM
DCX	3	17.14	4.68
Ki67	4	6.97	1.18
SOX1	5	11.12	5.22
SOX2	3	9.64	3.59
Nestin	3	7.06	4.10
Tuj1	3	77.21	1.91
**8 weeks**			
Antibody name	n	Mean %	SEM
DCX	4	3.65	1.14
Ki67	4	1.37	0.36
SOX1	4	1.68	0.72
SOX2	4	2.79	1.19
Nestin	4	1.89	0.73
Tuj1	3	71.53	22.15

This is an Open Access article distributed under the terms of the Creative Commons Attribution 4.0 International license (http://creativecommons.org/licenses/by/4.0/), which permits unrestricted use, distribution and reproduction in any medium, provided the original work is appropriately cited.

doi:10.14573/altex.1609122s1

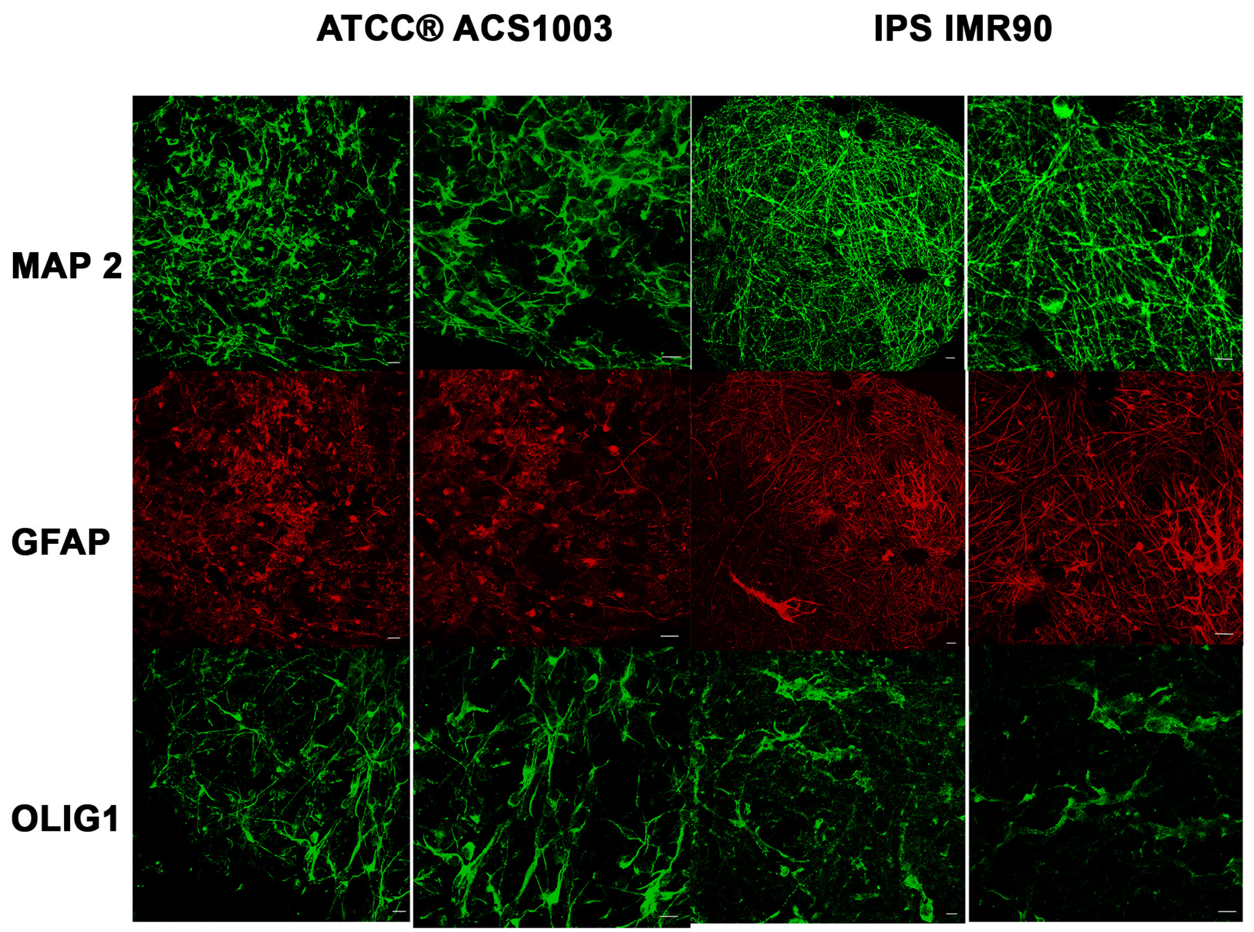

Fig. S1: IPS IMR90 and DYP0730 derived iPSC
MAP2 (neurons), GFAP (astrocytes) and OLIG1 (oligodendrocytes) immunohistochemistry characterization.

Video
Video at doi:10.14573/altex.1609122s2
